# A heat-inactivated H7N3 vaccine induces cross-reactive cellular immunity in HLA-A2.1 transgenic mice

**DOI:** 10.1186/s12985-016-0513-7

**Published:** 2016-03-31

**Authors:** Giuseppina Di Mario, Bruno Garulli, Ester Sciaraffia, Marzia Facchini, Isabella Donatelli, Maria R. Castrucci

**Affiliations:** Department of Infectious, Parasitic and Immune-Mediated Diseases, Istituto Superiore di Sanità, Viale Regina Elena, 299, 00161 Rome, Italy; Department of Biology and Biotechnology “Charles Darwin”, Sapienza University of Rome, 00185 Rome, Italy

## Abstract

**Background:**

Cross-reactive immunity against heterologous strains of influenza virus has the potential to provide partial protection in individuals that lack the proper neutralizing antibodies. In particular, the boosting of memory CD8+ T cell responses to conserved viral proteins can attenuate disease severity caused by influenza virus antigenic variants or pandemic strains. However, little is yet known about which of these conserved internal antigens would better induce and/or recall memory CD8+ T cells after in vivo administration of an inactivated whole virus vaccine.

**Methods:**

We explored the CD8 + T cell responses to selected epitopes of the internal proteins of an H7N3 influenza virus that were cross-reactive with A/PR/8/34 virus in HLA-A2.1 transgenic (AAD) mice.

**Results:**

CD8+ T cells against dominant and subdominant epitopes were detected upon infection of mice with live H7N3 virus, whereas immunization with non-replicating virus elicited CD8+ T cell responses against mostly immunodominant epitopes, which were rapidly recalled following infection with A/PR/8/34 virus. These vaccine-induced T cell responses were able to reduce the lung viral load in mice challenged intranasally with the heterologous influenza virus.

**Conclusions:**

A single immunization with non-replicating influenza virus vaccines may be able to elicit or recall cross-reactive CD8+ T cell responses to conserved immunodominant epitopes and, to some extent, counteract an infection by heterologous virus.

## Background

Influenza A virus pandemics continue to occur sporadically in the human population. Moreover, the emergence of a novel avian-origin reassortant influenza A (H7N9) virus in China with several characteristic features of mammalian influenza viruses poses concern for public health [1]. Currently, vaccination using inactivated influenza virus preparations remains the primary method of prevention, especially for pre-pandemic vaccines. In particular, A/PR/8/34 (PR8)-based pre-pandemic vaccine candidates, which contain the internal gene segments of PR8 virus and the HA and NA genes from avian influenza viruses with pandemic potential, have been generated and in some cases improved with respect to their growth characteristics [2, 3]. However, while vaccine-elicited antibodies will fully protect against homologous virus infection, eventually they offer only marginal protection against heterologous drift virus infection.

Although heterosubtypic immunity in humans appears to be weak, there is evidence that cross-reactive cell-mediated immune responses contribute to disease control [4–8]. Thus, vaccine-induced primary and memory responses by T cells against the relatively conserved internal antigens of influenza could play an important role in reducing the severity of disease caused by antigenic variants that could arise [9–13]. Although non-replicating influenza virus-based vaccines are known to be poor inducers of cytotoxic T cell (CTL) responses, advances in the field of adjuvants, delivery systems, and novel vaccination strategies hold promise for improving their potential effectiveness [14–17]. Inactivated whole virus vaccines with intact membrane fusion activity are known to facilitate cross-presentation to CD8+ T cells by antigen presenting cells (APC), and are more immunogenic than split vaccines [18, 19]. Moreover, gamma-ray-inactivation of whole-virus vaccines has been reported to preserve antigenicity, as well as cellular and humoral immunogenicity, to a greater extent than current viral inactivation methods [20]. Despite the important role that multiple antigenic determinants of internal viral proteins may have in protection against infection, little is yet known about which of these antigens would better induce and/or recall memory CD8+ T cells after in vivo administration of an inactivated whole virus vaccine.

Because of different HLA haplotypes and MHC Class I-dependent CD8+ T cell functions, the quality and quantity of a T cell response can vary greatly between individuals. HLA-A*0201 is an allele expressed by nearly half of the world’s population and several studies have been performed to determine the HLA-A2-restricted CD8+ T cell responses following natural influenza virus infection [21, 22]. In the present study, we investigated the HLA-A*0201-restricted epitope specificities of CD8+ T cells in HLA-A2.1 transgenic (AAD) mice responding to a non-replicating H7N3 whole virus, and revealed the breadth of recall responses following infection of these mice with heterologous PR8 virus. We also determined whether these vaccine-induced virus-specific CD8+ T cells, which target the internal conserved viral proteins, affected virus replication in the lungs of the mice.

## Methods

### HLA-A2.1 transgenic mice

AAD mice were obtained from The Jackson Laboratory (Bar Harbor, ME, USA). These mice with the C57BL/6J genetic background express an interspecies hybrid class I molecule, composed of the alpha 1 and alpha 2 domains of the human HLA-A*0201 allele and the alpha 3, cytoplasmic and transmembrane domains of the mouse H-2D^d^ class I molecule [23]. Female mice were used at 8–10 weeks of age; genetic authenticity was confirmed by performing PCR for the transgene. All mice were maintained at Istituto Superiore di Sanità following institutional guidelines and were used under approved protocols.

### Peptides

The H2-D^b^ restricted NP_366_ peptide and thirteen peptides that bind the HLA-A2.1 molecule and are conserved among diverse influenza subtypes were synthesized by Primm (Italy). Peptide purity was >90 % in all cases, and the identity of the peptides was verified by using spectrometry. The peptides were dissolved in DMSO at 10 mg/ml and stored at -20 °C.

### Generation of PR8-NP_N370Q_ virus

The mutant recombinant virus PR8-NP_N370Q_, in which the asparagine at the fifth position of the epitope was replaced with glutamine (N370Q), was generated by using plasmid-driven reverse genetics, as described by Neumann et al. [24]. The presence of the NP mutation was confirmed by sequencing PCR-amplified cDNA.

### Immunization of mice and challenge experiments

Groups of AAD mice were injected intraperitoneally (i.p.) with 500 HAU (approximately 3.5 μg of viral protein in a 0.5-ml volume) of either live or heat-inactivated (HI) low pathogenic A/turkey/Italy/214845/02 virus (HI-H7N3). Heat inactivation of small volumes of virus suspension was performed in a water bath at 56 °C for 30 min and confirmed by the complete loss of infectivity of MDCK cells. Intranasal (i.n.) immunization was performed by anesthetizing mice with Avertin (2,2,2-tribromoethanol) and then inoculating them with 10^2.4^ TCID_50_ of PR8 virus, resuspended in 40 μl of PBS. For the viral challenge, mice in groups of 7 were i.n. infected with 3 LD_50_ (50 % lethal dose), corresponding to 10^3.7^ TCID_50_ in a volume of 40 μl, of PR8-NP_N370Q_ virus. Naïve mice were included as a negative control. The mice were weighted daily and monitored for mortality for two weeks. To determine viral lung titers, mice were sacrificed 3 and 7 days post-infection (p.i.), and lung homogenates were titrated in MDCK cells. The limit of viral detection was 0.5 log_10_ TCID_50_/ml.

### IFN-γ ELISPOT assays

Spleens of mice (5–7 mice per group) were collected at the indicated time points and assayed for antigen-specific IFN-γ-producing cells by using an IFN-γ ELISPOT assay. In the challenge experiments, inflammatory cells were also collected from the lymph nodes draining the respiratory tracts (mediastinal lymph nodes, MLN), lungs and bronchoalveolar lavages (BAL) of infected mice as previously described, and then directly subjected to the ELISPOT assay [25, 26]. Single-cell suspensions from the lymphocytic populations were cultured with the indicated synthetic peptides or DMSO in anti-IFN-γ-coated plates at 37 °C for 36–40 h. Colored spots representing IFN-γ-releasing cells are reported as the number of spot-forming cells (SFC) per 10^6^ cells.

### Serology

Serum samples were collected from mice vaccinated with HI-H7N3 immediately before and 7 days after challenge, and tested for the presence of influenza-specific antibodies by using an hemagglutination–inhibition (HI) assay and a micro-neutralization (MN) assay, as described previously [27]. Sera were also tested in ELISA, using purified whole inactivated viruses [28].

### Statistics

The statistical significance of the differences between groups of animals was determined by using the Wilcoxon-Mann-Whitney test; *P* < 0.05 was considered significant.

## Results

### Induction of influenza-specific CD8+ T cell responses in AAD mice following vaccination with H7N3 virus

The initial experiments were designed to determine the breadth and specificity of primary CD8+ T cell responses that were elicited against the internal viral proteins in AAD mice following i.p. immunization with the same dose of live or HI-H7N3 virus. To this end, a panel of influenza-derived CD8+ T cell epitope peptides (see Table 1), consisting of 1 nucleoprotein (NP) epitope, 10 polymerase epitopes, and 2 matrix (M1) epitopes, was used in IFN-γ ELISPOT assays because they are highly conserved among different influenza subtypes. These CD8+ T cell epitopes were previously reported to be generated by natural processing of influenza antigens in HLA-A2+ donors and were found to be immunogenic following subcutaneous injection of HLA-A2/K^b^ transgenic mice [21, 22, 29].

**Table 1:** Influenza A virus-derived MHC class I restricted T cell epitopes included in the study

Peptides	Sequence	Position	MHC restriction
M1-58	GILGFVFTL	58–66	HLA-A2.1
M1-59	ILGFVFTLTV	59–68	HLA-A2.1
PB1-407	MMMGMFNML	407–415	HLA-A2.1
PB1-413	NMLSTVLGV	413–421	HLA-A2.1
PB1-501	FVANFSMEL	501–509	HLA-A2.1
PB1-505	FSMELPSFGV	505–514	HLA-A2.1
PB2-49	WMMAMKYPI	49–57	HLA-A2.1
PB2-50	MMAMKYPITA	50–59	HLA-A2.1
PA-46	FMYSDFHFI	46–54	HLA-A2.1
PA-86	RTMAWTVVNSI	86–96	HLA-A2.1
PA-225	SLENFRAYV	225–233	HLA-A2.1
PA-282	FLLMDALKL	282–290	HLA-A2.1
NP-329	QLVWMACHSAA	329–339	HLA-A2.1
NP-366	ASNENMETM	336–374	H2-D^b^

**Fig. 1:**
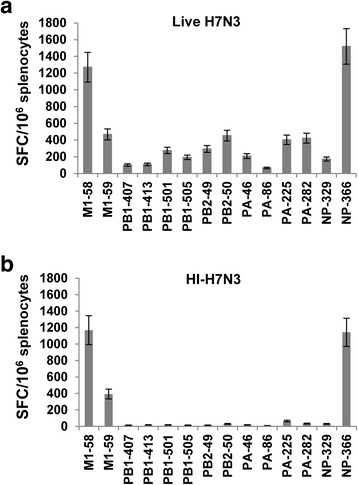
HLA-A2-restricted influenza-specific CD8 + T cell responses in AAD mice. Groups of AAD mice (5/group) were immunized i.p. with 500 HAU of live (**a**) or heat-inactivated (HI)-H7N3 virus (**b**). Twelve days later, influenza-specific CD8+ T cell responses were measured in cells from freshly isolated splenocytes of individual mice by use of an ex vivo IFN-γ-ELISPOT assay with the indicated peptides. Bars represent means ± standard deviation (SD) for five mice per group. The data are representative of three independent experiments that gave similar results

Although the intramuscular or intranasal route is used for vaccination in humans, the i.p. route of viral inoculation does not permit productive influenza replication in mice, and it is used to examine CD8+ T cell responses in many studies performed in murine models of influenza virus infection [30–32]. When mice were injected i.p. with the live H7N3 virus, all the selected peptides containing dominant and subdominant T cell epitopes stimulated IFN-γ secretion from mouse splenocytes in ex vivo ELISPOT assays (Fig. 1a). In particular, a strong CD8+ T cell response to the HLA-A2-restricted immunodominant M1_58_ epitope was observed in all mice. This is in accordance with previous studies in AAD mice showing that CTL recognition of the M1_58_ peptide in the context of the alpha 1 + alpha 2 domains was greatly augmented by replacing the human alpha 3 domain with its murine counterpart in the transgenic class I molecule [23]. The immunodominant peptide epitope NP_366_ was also included in the assay to document the H2-D^b^-restricted CD8+ T cell response that has always been observed in these AAD transgenic mice with the C57BL/6J genetic background [33]. Interestingly, the responses specific to peptide epitope H2-D^b^-PA_224_ were weaker in these mice compared with the co-dominant CD8 reactivity with the H2-D^b^-NP_366_ epitope that has previously been measured in C57BL/6J mice following primary virus infection (data not shown and [34]). Similar reactivity to H2-D^b^-PA_224_ was also measured with the overlapping PA_225_ peptide epitope, which was recognized by memory CD8+ T cells in A2+ healthy donors [22]. For this reason, the murine peptide epitope H2-D^b^-PA_224_ was not included in the analysis.

In contrast to the live viral infection, mice immunization with HI-H7N3 virus, which maintains fusion activity, elicited CD8+ T cell responses that were mainly directed at immunodominant epitopes M1_58_ and H2-D^b^-NP_366_. Reproducible T cell responses were also observed for the overlapping peptide M1_59_, whereas undetectable or lower numbers (50–80 SFC/10^6^ cells) were consistently measured for the other subdominant epitopes, compared with those measured in mice infected with live virus (Fig. 1b).

Together, these data indicate that a broad repertoire of CD8+ T cell specificities are elicited in AAD mice upon i.p. injection of live influenza virus, and that vaccination with non-replicating H7N3 virus narrows the immune response to the immunodominant epitopes.

### Induction of CD8+ T cell recall responses to internal viral proteins in mice immunized with HI-H7N3 virus and then infected with PR8 virus

**Fig. 2:**
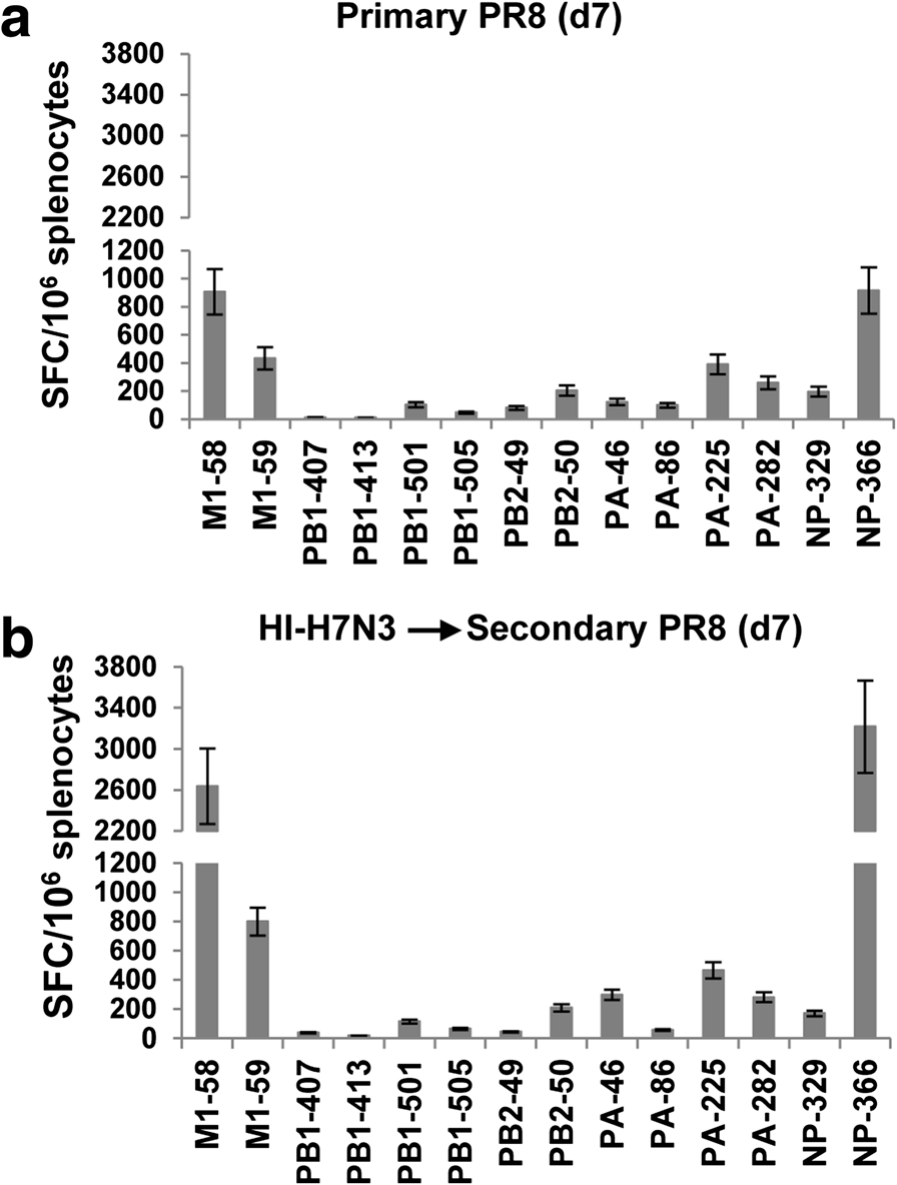
HLA-A2-restricted influenza recall responses in mice immunized with HI-H7N3 virus and then infected with PR8 virus. Naïve mice (**a**) or mice vaccinated with HI-H7N3 virus 4 weeks earlier (**b**) (5/group) were challenged i.n. under anesthesia with 10^2.4^ TCID_50_ of PR8. Seven days after challenge, mice were sacrificed and influenza-specific CD8+ T cell responses were measured in cells from freshly isolated splenocytes of individual mice by means of an ex vivo IFN-γ-ELISPOT assay with the indicated peptides. Bars represent means ± SD for five mice per group. The data are representative of two independent experiments that gave similar results

To further investigate the immunogenicity of whole inactivated virus vaccines, mice were vaccinated with HI-H7N3 virus and, one month later, i.n. infected with a sublethal dose of PR8 virus. Then, virus-specific CD8+ T cell responses were examined on day 7 p.i. by measuring IFN-γ production from splenocytes in an ELISPOT assay in the presence of the above-mentioned peptides. Primary infection of control mice with PR8 virus induced CD8+ T cell responses to the immunodominant H2-D^b^-NP_366_ and M1_58_ epitopes and to other HLA-A2 restricted subdominant epitopes (Fig. 2a). When mice were vaccinated with HI-H7N3 prior to PR8 virus infection, the frequencies of immunodominant epitopes increased at least 3-fold in the spleens, whereas the frequencies of the subdominant epitopes were similar to those observed in unvaccinated mice (Fig. 2b).

These results provide further evidence that HI-H7N3 virus could prime CD8+ T cell responses specific to the immunodominant T cell epitopes that were rapidly recalled following viral infection.

### Induction of influenza-specific CD8+ T cell responses by vaccination with HI-H7N3 virus in the presence of pre-existing immunity to influenza virus

**Fig. 3:**
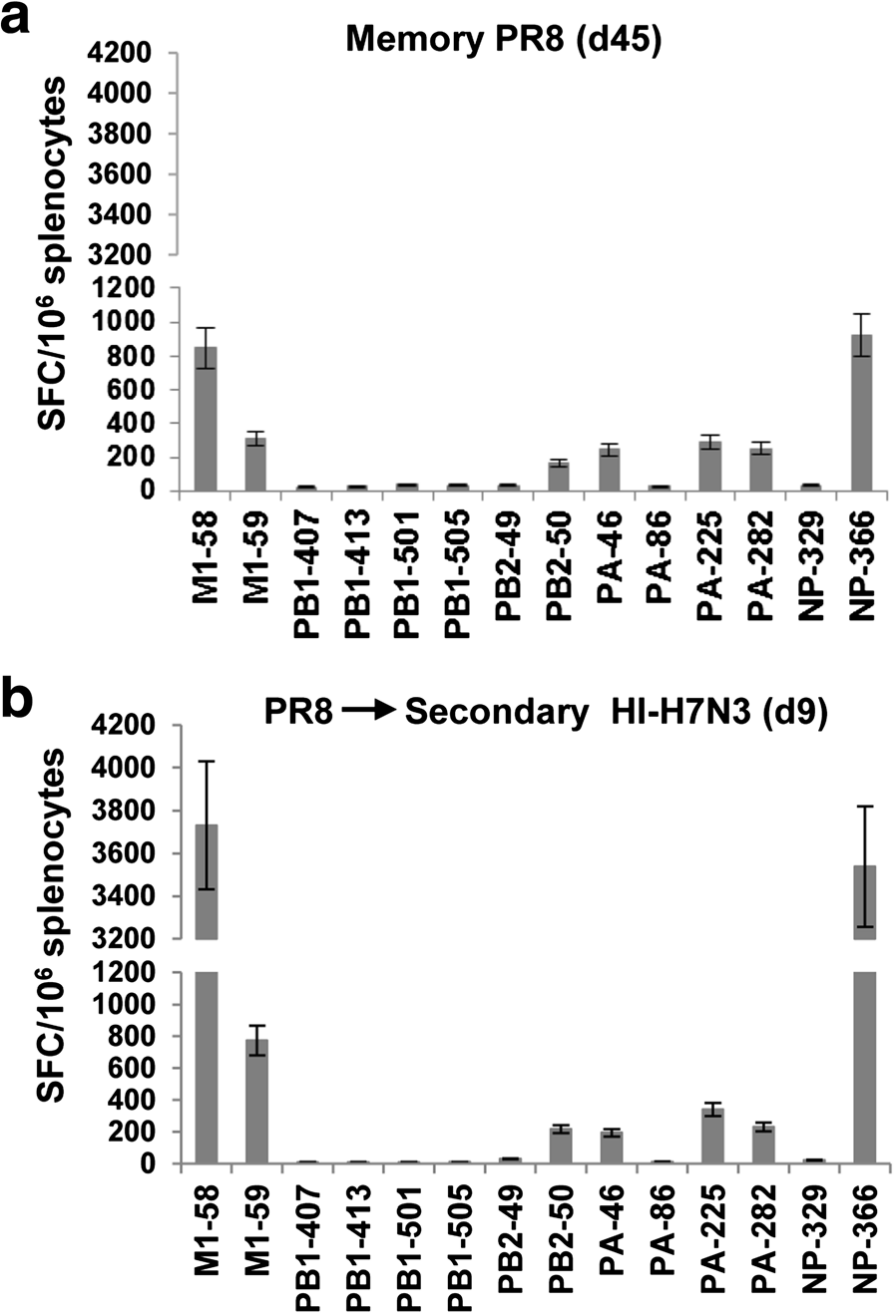
Frequency of HLA-A2-restricted influenza specific CD8+ T cells in AAD mice with pre-existing immunity to PR8 virus. Groups of naïve mice (7/group) were infected i.n. under anesthesia with 10^2.4^ TCID_50_ of PR8 virus. Six weeks later, they were injected i.p. with PBS (**a**) or 500 HAU of HI-H7N3 virus (**b**). Nine days after vaccination, the mice were sacrificed and influenza-specific CD8+ T cell responses were measured in cells from freshly isolated splenocytes of individual mice by using an ex vivo IFN-γ-ELISPOT assay with the indicated peptides. Bars represent means ± SD for 7 mice per group. The data are representative of three independent experiments that gave similar results

We next sought to determine whether prior exposure of mice to a heterologous influenza virus affected the immunogenicity of the HI-H7N3 vaccine. Groups of mice were i.n. primed with sublethal doses of PR8 virus, and then six weeks later vaccinated i.p. with the HI-H7N3 virus. As shown in Fig. 3, the HI-H7N3 virus boosted the CD8+ T cell responses specific to the immunodominant epitopes in the presence of pre-existing immunity to influenza. Specifically, a 3- to 5-fold increase was measured for the dominant M1_58_ and H2-D^b^-NP_366_ peptides, relative to the mice that received only the PR8 virus, whereas the frequency of the immune effectors specific to the subdominant epitopes was at a similar low level in both groups of mice, regardless of pre-existing host immunity to the virus. Overall, these data show that the inactivated whole virus vaccine could induce recall responses to the H2-D^b^-NP_366_ and M1_58_-specific epitopes.

### Virus load in the lungs of HI-H7N3 virus-vaccinated mice upon challenge with a heterologous influenza virus

**Fig. 4:**
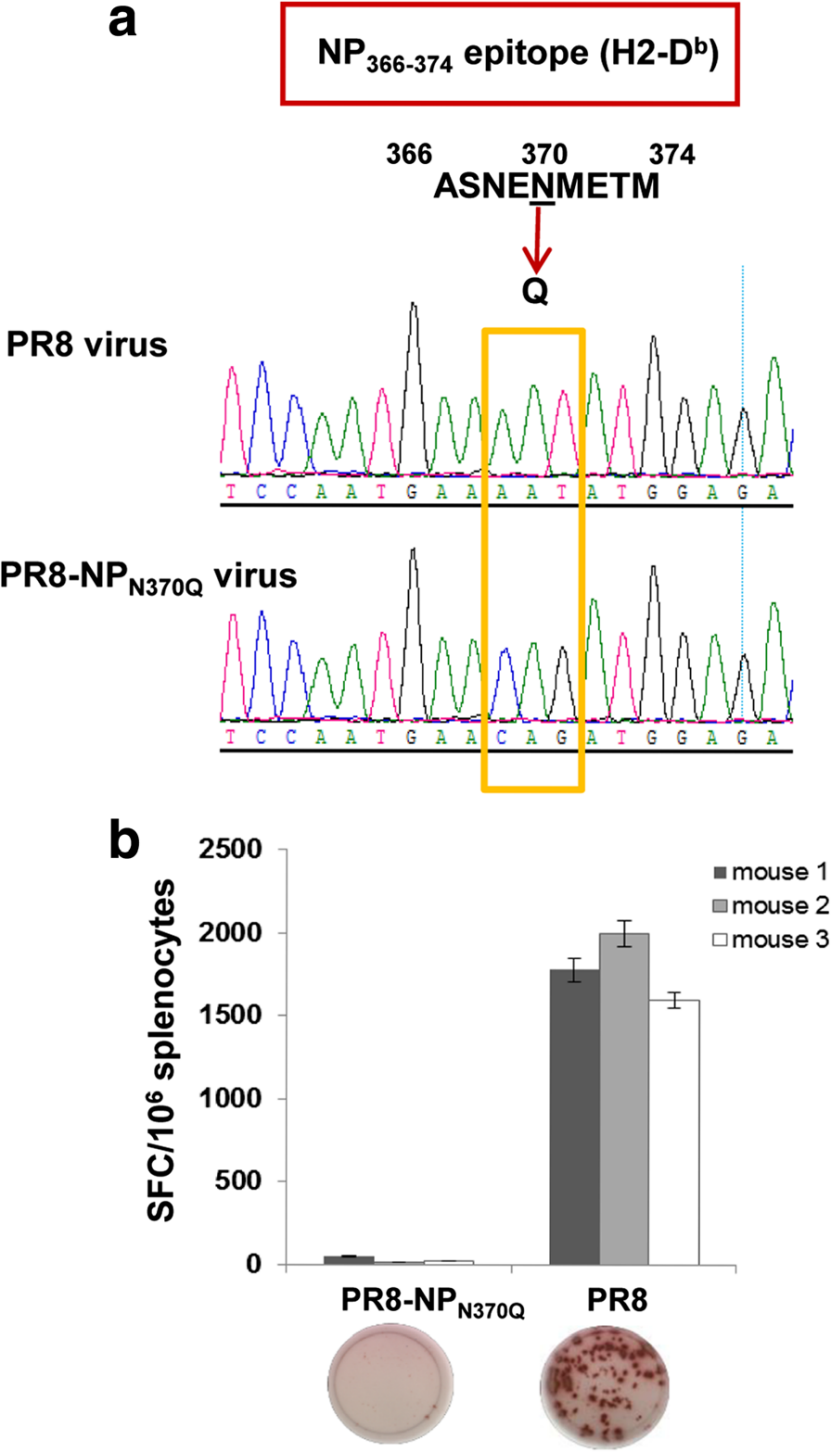
Amino acid mutation in the H2-D^b^-NP_366_ epitope and CD8+ T cell recognition following virus infection in AAD mice. **a** Sequence electropherograms showing the change from Asparagine (N) to Glutamine (Q) at position 370 in the H2-D^b^-NP_366_ epitope of the PR8-NP_N370Q_ virus. **b** Mice were infected i.n. with 10^2.4^ TCID_50_ of PR8 or PR8-NP_N370Q_ virus. Nine days later, the mice were sacrificed and the frequency of H2-D^b^-NP_366_-specific IFN-γ-producing T cells was measured by using an ELISPOT assay on spleen-derived lymphocytes from individual mice. Results are expressed as means of triplicate wells ± SD. The stained wells of representative samples are shown below the graphical representations of the number of spot forming units (SFU) per 10^6^ cells

**Fig. 5:**
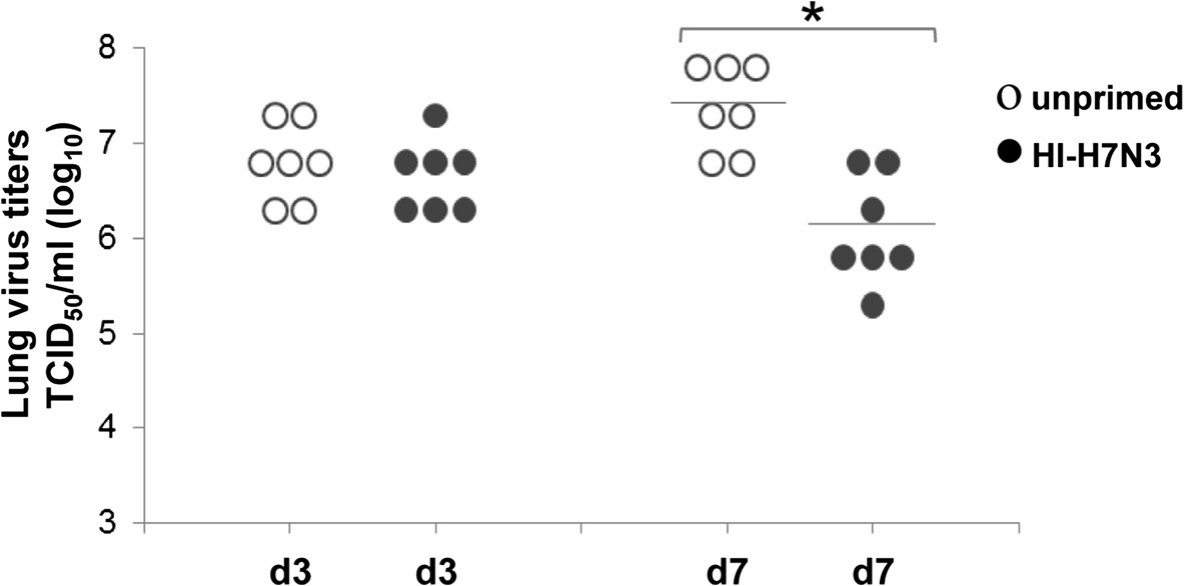
Viral load levels in the lungs of mice following infection with PR8-NP_N370Q_ virus. Groups of mice (7/group) immunized with the HI-H7N3 virus were infected i.n. four weeks later with 3 LD_50_ (which corresponds to 10^3.7^ TCID_50_) of PR8-NP_N370Q_ virus. Naïve mice inoculated with PR8-NP_N370Q_ virus served as controls. Three days and seven days after challenge, mice were sacrificed and lungs were collected for viral titration on MDCK cells. Virus titers from individual mice are presented as log_10_ TCID_50_ per ml (expressed as the mean ± SD log_10_TCID_50_/g of tissue). One of three similar experiments is shown. *P < 0.005 compared with unprimed mice

**Fig. 6:**
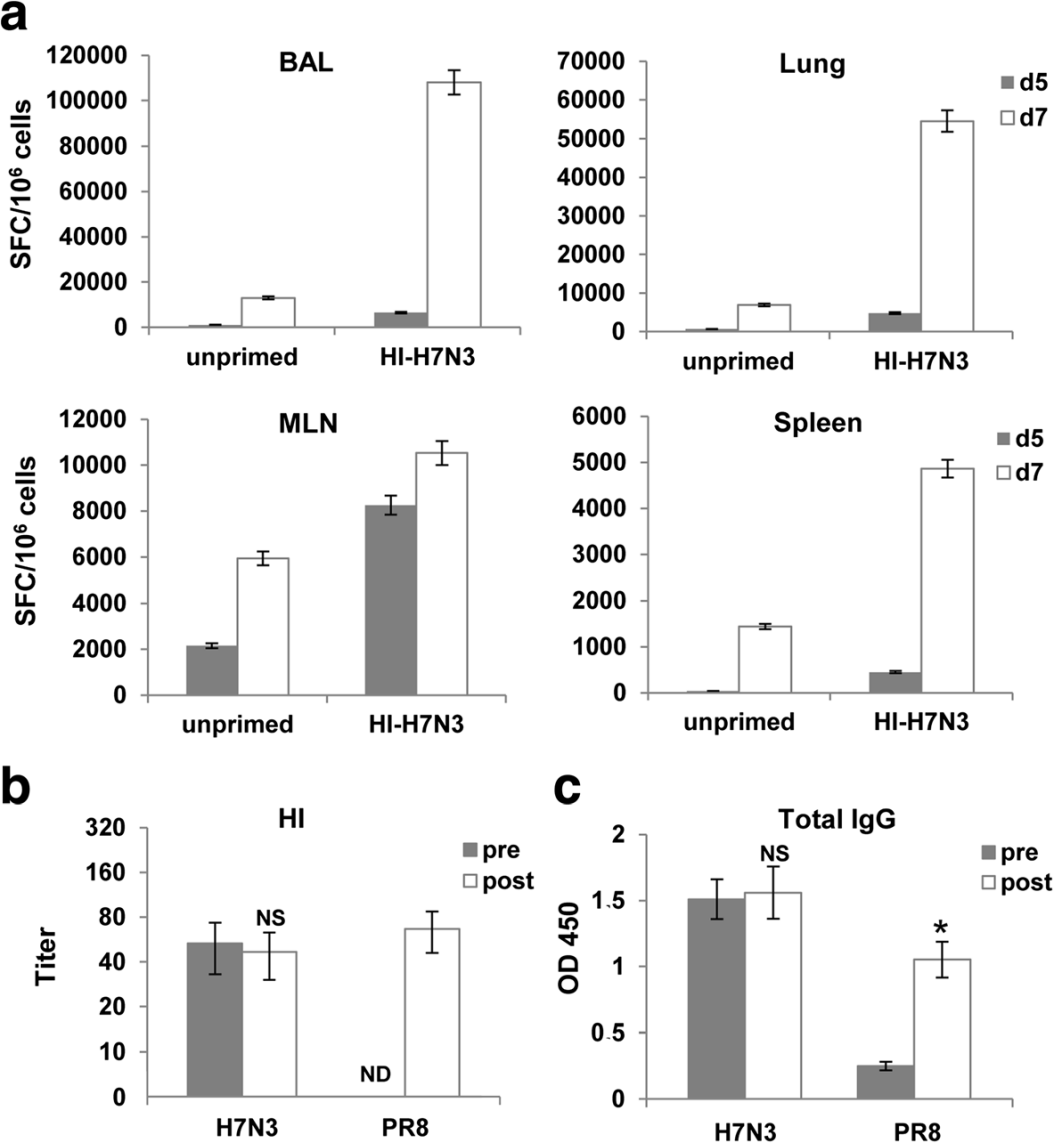
Quantification of M1_58_-specific CD8 + T cells isolated from lung airways and lymphoid organs, and influenza-specific antibodies following PR8-NP_N370Q_ challenge. Groups of mice were treated as described in the legend for Fig. 5. **a** On day 5 or day 7 post-challenge, the mice were sacrificed and the numbers of M1_58_-specific CD8+ T cells were examined in the BAL, lungs, MLN, and spleen by using the IFN-γ-ELISPOT assay. Results are expressed as means of triplicate wells ± SD. **b** & **c** Serum samples were drawn before (pre) and 7 days post-challenge (post) from mice previously immunized with the HI-H7N3 virus, and specific antibodies against H7N3 and PR8 viruses were measured by HI (**b**) and ELISA (**c**); ELISA for IgG antibodies; values for sera were obtained with use of 100-fold-diluted samples. ND = None detected. OD 450 = Optical density at 450 nm. Values represent mean of 6 mice per group ± SD. One of three similar experiments is shown. *P < 0.05, NS = not significant, compared with pre-challenge sera

Previous studies have shown the protective efficacy of M1_58_-specific CD8+ T cells against virus infection in HLA-A2 mice [35, 36]. Our results suggest that the CD8 + T cell cross-reactivity between the internal proteins of PR8 virus and the non-replicating HI-H7N3 vaccine in AAD mice involves the M1_58_ and H2-D^b^-NP_366_ epitopes. To evaluate whether the M1_58_-specific T cells elicited by HI-H7N3 may play a role in protection against challenge virus replication, we generated the recombinant influenza virus PR8-NP_N370Q_, in which the anchor residue of the H2-D^b^-NP_366_ epitope that binds the murine H2-D^b^ MHC-I glycoprotein is mutated. This mutation results in the absence of H2-D^b^-NP_366_ complexes and a lack of CD8+ T cell recognition, that correlates with a more severe disease, in spite of comparable lung virus titers with those of mice infected with the wild-type PR8 virus [31] (Fig. 4). We used this virus to investigate whether cross-reactive CD8+ T cells could limit the infection in mice vaccinated with HI-H7N3 virus and subsequently challenged with lethal doses of PR8-NP_N370Q_ virus. A single dose of a parenterally administered vaccine was not capable to improve mouse survival and weight loss in this viral challenge model (data not shown). Nevertheless, the viral load in the lungs of the immunized mice was reduced at 7 days post-challenge by 20-to 40-fold compared with the control mice (Fig. 5). A vigorous recall response of CD8+ T cells targeting the M1_58_ epitope was measured in the spleen of these mice compared with the non-immunized mice. Moreover, the recruitment of high levels of antigen-driven IFN-γ-producing CD8+ cells to the airways and infected lung by day 5 post-challenge suggests that the cross-reactive M1_58_-specific CD8+ T cells were likely responsible for the reduced lung viral loads (Fig. 6a).

To exclude the possibility that cross-reactive antibodies could also have contributed to this viral control, pre-challenge sera obtained from mice that received the HI-H7N3 vaccine, and post-challenge sera were analyzed for the presence of influenza-specific antibodies. After immunization with HI-H7N3 vaccine, mice developed virus-specific antibodies that were detected by HI assay that did not cross-react with PR8 virus (Fig. 6b). Similar results were observed in the MN assay (data not shown). When tested against detergent-disrupted viruses by ELISA, pre-challenge sera as well as post-challenge sera showed similar antibody levels against the H7N3 virus. In contrast, only post-challenge sera showed high levels of reactivity against the PR8 virus (Fig. 6c).

Overall, our results show that cross-protective cellular immune responses can be induced in AAD mice following systemic immunization with an inactivated whole-virus influenza vaccine.

## Discussion

In the present study, we evaluated CD8 + T cell responses to internal viral proteins elicited by an inactivated H7N3 virus in AAD mice. We found that CD8+ T cell responses to both the immunodominant epitopes and to most of the highly conserved subdominant epitopes could be detected in AAD mice upon inoculation with live virus, whereas immunization with HI-H7N3 virus induced CD8+ T cell responses mainly to the immunodominant epitopes. We also found that the vaccine-induced T cell responses appeared to reduce the lung viral load in mice challenged with the PR8-NP_N370Q_ virus, which lacks the H2-D^b^-NP_366_ epitope.

Most influenza-specific memory T cells that are detectable in humans are directed mainly against the remarkably conserved epitopes of the internal viral proteins, and have been reported to accumulate either as resident or central memory T cells in the lungs and lymphoid tissues, respectively, following recovery from influenza virus infections [37–41]. Therefore, the rapid boosting of these cross-reactive T cells in the respiratory tract by a pre-pandemic vaccine could be extremely beneficial to provide protection against severe influenza virus infections [42]. Here, the CD8 + T cell responses to the HLA-A2-restricted influenza epitope M1_58_ paralleled those specific to the H2-D^b^-NP_366_ epitope in AAD mice, following immunization with live virus. Interestingly, the level of responses specific to the H2-D^b^-PA_224_ epitope was lower in AAD mice than in C57BL/6 mice. This low reactivity may reflect a reduced level of antigen-specific T cell precursors in AAD mice, compared with the higher frequency of naïve precursors for H2-D^b^-PA_224_ than H2-D^b^-NP_366_ in C57BL/6 mice [43]. Alternatively, it may be due to superior competition of T cells specific to the H2-D^b^-NP_366_ and M1_58_ epitopes from the most abundant internal proteins during antigen presentation.

In AAD mice immunized with a single dose of inactivated H7N3 vaccine, CD8+ T cell responses were mainly elicited by immunodominant epitopes. Several factors contribute to the differences between live and inactivated viruses in the CD8+ T cell response to the subdominant epitopes. In particular, the provision of additional antigens in the form of endogenously synthesized viral proteins, following infection with a live virus, plays a key role. Our results are consistent with those of other studies showing that epitopes derived from more abundant viral proteins contribute to immunodominant responses, although the inherent features of the antigens and their capacity to be cross-presented by APC also likely play a role [32, 44–46]. Likewise, others have reported that CTL induction by cross-presentation of Lymphocytic Choriomeningitis virus antigens, that were constitutively expressed in tumor cell lines and injected in mice, was driven by immunodominant but not subdominant epitopes [47]. To further investigate the immunogenicity of the whole inactivated virus vaccine, we used HI-H7N3 in a prime-boost approach with live preparations of serologically distinct PR8 virus. We detected and efficiently recalled, upon subsequent viral infection, mainly CD8+ T cell responses that were restricted to the immunodominant epitopes H2-D^b^-NP_366_ and M1_58_, and the overlapping M1_59_. Importantly, the M1_58_-specific CD8+ T cells were efficiently recruited to the respiratory tract by subsequent i.n. infection with PR8-NP_N370Q_ virus, and were likely responsible for the reduction in lung viral load. Although we cannot exclude that CD4+ and CD8+ T cells with other specificities may help reduce viral infection, the lack of serum cross-reactive antibodies further corroborates the most likely protective effect of M1_58_-specific CD8+ T cells. Nevertheless, no changes were observed in mortality of these mice receiving a single dose of a parenterally administered vaccine as compared to unvaccinated mice. Our results agree with other research findings showing that a subsequent influenza virus infection in the respiratory tract is required for reactivation of i.p. primed influenza virus-specific CD8+ T cells and access to the lung airways [38, 48]. Indeed, activation and maintenance of T cell populations in the airways are dependent upon the route of infection and prolonged presentation of viral antigens in the lymph nodes draining the respiratory tract. Thus, new immunization strategies that induce broadly cross-reactive antibodies and T-cell responses at mucosal surfaces could greatly improve influenza vaccine efficacy. Although mouse models do not closely reflect human influenza virus infection, yet our data suggest that a single immunization with a whole inactivated influenza virus vaccine might also be able to elicit or recall cross-protective immune responses specific to immunodominant NP and M epitopes in humans, and thus, to some extent, counteract an infection by an heterologous virus. In this context, it would be interesting to explore the immunogenic pattern and magnitude of the response elicited in the presence of adjuvants or following immunization of these mice with live, attenuated influenza vaccine (LAIV). Previous studies have shown quantitative and qualitative differences in T cell responses to LAIV and inactivated vaccines. In particular, viral replication of LAIV in the respiratory mucosa, especially of young children with low levels of pre-existing immunity, provides better protection against infection, likely due to a broader array of dominant and subdominant T cell responses as well as beneficial induction of immune-effectors at the site of infection [49, 50].

The M1_58_ epitope is highly conserved among multiple influenza viral strains and subtypes, and it is able to elicit CTL responses in both HLA-A*02- and HLA-C*08-positive individuals [29, 51]. Although functional constraints on influenza virus CTL epitopes may limit escape from CTL-mediated immunity, epitope mutations may occur and, in some cases, these variant epitopes can still be recognized, to some extent, by cross-reactive T cells [52–54]. Indeed, CTL escape variants have been shown to arise following independent amino acids mutations either at the anchor residues or in the T cell receptor contact residues of CTLs specific to the NP of influenza A viruses isolated during circulation in human populations [55, 56]. For these reasons, it is strongly desirable to have more epitopes recognized by the immune system to fight a viral infection. Furthermore, we cannot exclude the possibility that inactivated whole virus-based vaccines of avian origin would cross-react better than PR8-based vaccines against new emerging zoonotic influenza viruses in human hosts. Although viral internal proteins are highly conserved, the 10 %–15 % sequence dissimilarity in the NP of different influenza subtypes, for example, may be responsible for the loss of epitopes of unknown HLA restrictions. Therefore, in the context of inactivated vaccines against highly pathogenic influenza strains that could arise from animal reservoirs, the recall of dominant CD8+ T cell responses targeting the M and NP proteins most closely related to the emerging virus strains could be of great value in vaccine effectiveness. Further studies are warranted to better characterize the relationship between pre-existing CD8+ T-cell immunity across the multiple ethnicities established in the human population and epitope conservancy among the internal proteins of potentially pandemic influenza strains [57].

## Conclusion

In summary, our data show that a non-replicating whole virus-based vaccine elicited cross-reactive influenza virus-specific CD8+ T cell responses that were restricted predominantly to immunodominant epitopes, and that substantial numbers of effector-memory T cells were recruited into the lungs of AAD mice for protection against heterologous virus infection. Vaccination strategies designed to improve the induction of or to boost immune responses to the most conserved epitopes of newly emerging influenza A virus strains would be extremely useful for the development of pre-pandemic vaccines.
